# Computational Framework of Magnetized MgO–Ni/Water-Based Stagnation Nanoflow Past an Elastic Stretching Surface: Application in Solar Energy Coatings

**DOI:** 10.3390/nano12071049

**Published:** 2022-03-23

**Authors:** Muhammad Mubashir Bhatti, Osman Anwar Bég, Sara I. Abdelsalam

**Affiliations:** 1 College of Mathematics and Systems Science, Shandong University of Science and Technology, Qingdao 266590, China; 2 Multi-Physical Engineering Sciences Group, Mechanical Engineering, School of Science, Engineering and Environment (SEE), Salford University, Manchester M5 4WT, UK; o.a.beg@salford.ac.uk; 3 Basic Science, Faculty of Engineering, The British University in Egypt, Al-Shorouk City 11837, Egypt; sara.abdelsalam@bue.edu.eg

**Keywords:** MgO–Ni nanoparticles, magnetic hybrid nanofluids, porous medium, thermal and velocity slip, solar coatings, stagnation flow

## Abstract

In this article, motivated by novel nanofluid solar energy coating systems, a mathematical model of hybrid magnesium oxide (MgO) and nickel (Ni) nanofluid magnetohydrodynamic (MHD) stagnation point flow impinging on a porous elastic stretching surface in a porous medium is developed. The hybrid nanofluid is electrically conducted, and a magnetic Reynolds number is sufficiently large enough to invoke an induced magnetic field. A Darcy model is adopted for the isotropic, homogenous porous medium. The boundary conditions account for the impacts of the velocity slip and thermal slip. Heat generation (source)/absorption (sink) and also viscous dissipation effects are included. The mathematical formulation has been performed with the help of similarity variables, and the resulting coupled nonlinear dimensionless ordinary differential equations have been solved numerically with the help of the shooting method. In order to test the validity of the current results and the convergence of the solutions, a numerical comparison with previously published results is included. Numerical results are plotted for the effect of emerging parameters on velocity, temperature, magnetic induction, skin friction, and Nusselt number. With an increment in nanoparticle volume fraction of both MgO and Ni nanoparticles, the temperature and thermal boundary layer thickness of the nanofluid are elevated. An increase in the porous medium parameter (Darcy number), velocity slip, and thermal Grashof number all enhance the induced magnetic field. Initial increments in the nanoparticle volume fraction for both MgO and Ni suppress the magnetic induction near the wall, although, subsequently, when further from the wall, this effect is reversed. Temperature is enhanced with heat generation, whereas it is depleted with heat absorption and thermal slip effects. Overall, excellent thermal enhancement is achieved by the hybrid nanofluid.

## 1. Introduction

Renewable energy sources have become increasingly important in recent years, owing to the depletion of fossil fuels and the rise in the price of electricity. Furthermore, fossil fuels emit CO_2_, whereas renewable energy sources do not pose this problem and are sustainable and ecologically desirable. Environmentalists believe that the adoption of renewable energy sources will help to reduce global warming and greenhouse emissions [1]. Solar energy has emerged as a viable alternative source of renewable energy in recent years since it is easily accessible, free of pollutants, and causes the least amount of harm to the environment [2]. Solar power plants are capable of supplying thermal energy for use in household applications [3]. The twenty-first century accounts for 40% of the world’s fuel market and 60% of the world’s energy production. According to a recent study [4], worldwide CO_2_ emissions will drop by 75 percent by 2050, compared to 1985 levels. As a result, solar energy will play an important role and serve as a natural substitute. Following the process of solar energy conversion, electricity can be converted into thermal energy by using a steam turbine. However, conventional heat transfer demonstrates lower thermal conductivity, and as a result, small solid particles should be added to the base fluid in order to increase thermal conductivity. As a result, nanofluids play an important role in this application [5]. Choi and Eastman [6] were the first to use the term nanofluid, which he coined in 1995 in order to fill the gap. Nanofluids have gained significant prominence in recent years as a result of their remarkable thermal performance capabilities. The use of nanoparticles in renewable energy sources has the potential to significantly improve the heat transfer characteristics of existing devices [7]. Several authors have recently investigated the use of nanofluids in a variety of thermal engineering and renewables applications [8,9].

An entirely new class of nanofluid [10,11], referred to as hybrid nanofluid, has recently been discovered. A hybrid nanofluid can be manufactured by dispersing two or more types of nanoparticles in a base fluid. A hybrid material is a substance that combines the chemical and physical properties of several materials at the same time, resulting in a homogeneous phase. It is possible to achieve remarkable physicochemical properties in synthetic hybrid nanomaterials that are not possible with their individual components. A substantial number of studies have therefore been communicated to explore the properties of these composite [12] and hybrid nanomaterials made up of carbon nanotubes, which can be used in a variety of applications such as nanocatalysts, biosensors, solar collectors, coatings, and electrochemical sensors [13]. Devi and Devi [14] computed the hybrid nanofluid (containing aluminum oxide and copper nanoparticles suspended in water) flow from an extending surface. Ghadikolaei et al. [15] evaluated the impact of different nanoparticle shapes in the stagnation flow of copper–titanium oxide/water hybrid nanofluid. Hassan et al. [16] investigated the heat transport properties of a hybrid nanofluid, including copper and silver nanoparticles. The natural convection of hybrid nanofluid flow over a porous medium with a non-uniform magnetic and circular heater was investigated by Izadi et al. [17]. Using an Eyring–Powell fluid model, Riaz et al. [18] investigated the thermal performance of non-Newtonian hybrid nanofluids (with a selection of metallic and carbon nanoparticles) in a wavy channel. Puneeth et al. [19] used a Casson fluid to analyze a three-dimensional hybrid nanofluid flow with a modified Buongiorno’s model along a nonlinear stretching surface, considering blood as the base fluid and titania metallic oxide nanoparticles.

Since MgO and Ni nanoparticles (NPs) play an important role in real-world applications, they are specifically considered in the current investigation. MgO NPs are economically feasible, environmentally friendly, and of significant industrial significance due to their unique physicochemical behaviors, which include an outstanding refractive index, higher thermal conductivity, excellent corrosion resistance, physical strength, extraordinary optical transparency, stability, flame resistance, and mechanical strength [20,21,22]. These features make MgO NPs useful as catalysts in organic transformation, semiconducting materials, photocatalysts, sorbents for inorganic and organic pollutants from wastewater, refractory materials, solar coatings, and electrochemical biosensors [23,24]. It is well-known that the depletion of fossil fuels contributes to global warming by increasing the level of air pollution, which, in turn, causes the sea level to rise. The advent of batteries, solar cells, and fuel cells to replace fossil fuels as an alternative energy source has helped to alleviate this problem. They release water as a bi-product, and hydrogen is produced, which is a spectacular source of fuel and an amazing alternative form of carbon-based bi-products. Magnesium, similar to other metals, is essential for hydrogen storage and plays an important part in this process. When compared to other hydrides, magnesium NPs have a distinct advantage due to their abundance in large quantities in the earth’s crust, their ability to store more hydrogen, ecologically beneficial properties, and low cost. However, nickel nanoparticles (NiNPs) are also useful in a variety of disciplines, including magnetic materials [25], biomedicine [26], energy technology [27], catalytic systems [28], catalysts for CO_2_ hydrogenation [29], magnetic biocatalysts [30] and electronics. Due to the high reactivity, environmentally friendly characteristics, and operational simplicity of nickel nanoparticles, they have been found to also be useful in a variety of organic reactions. These include the reduction of ketones and aldehydes [31], chemo-selective oxidizing coupling of thiols [32], α−alkylation of methyl ketone [33], synthesis of stilbenes from alcohol via Wittig-type olefination [34], and also hydrogenation of olefins [35].

In light of the numerous applications of MgO–Ni NPs, the purpose of this study is to investigate theoretically the magnetohydrodynamic water-based hybrid nanofluid stagnation flow impinging on a porous elastic stretching surface in a porous medium, as a model of solar collector coating manufacture. Heat generation/absorption and thermal/velocity slip effects are included. As revealed by the literature study, it is well-known that hybrid nanofluids provide promising results when compared to unitary (single nanoparticle) nanofluids in many applications. The mathematical model developed comprises the mass, momentum, energy, and induced magnetic field equations with appropriate boundary conditions. In addition, the Darcy law is deployed for porous medium effects, and viscous dissipation is incorporated. Numerical solutions of the transformed, dimensionless nonlinear ordinary differential boundary value problem are obtained with Matlab via a shooting method. Extensive visualizations of velocity, temperature, magnetic induction, skin friction, and the Nusselt number are presented graphically and with tables for the impact of emerging parameters. Validation of Matlab solutions with previously reported results (special cases) is included. Detailed interpretation of the results is provided.

## 2. Magnetic Hybrid Nanofluid Stagnation Flow Model

**Figure 1:**
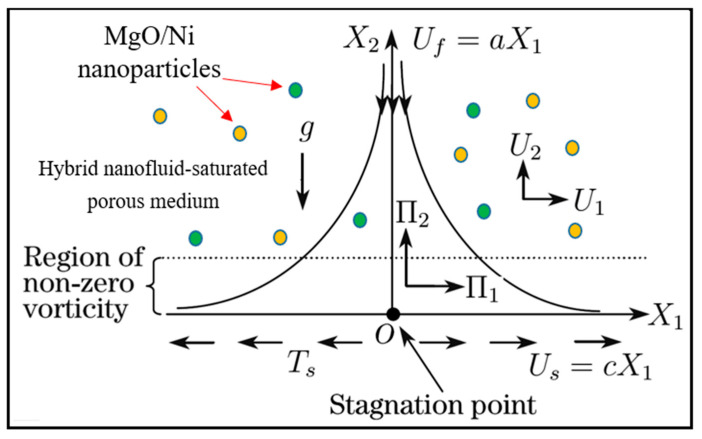
Two-dimensional geometrical configuration for magnetized hybrid nanofluid stagnation point flow over an elastic surface in a porous medium.

The physical regime under consideration comprises a steady orthogonal stagnation point flow of an incompressible hybrid magnetized nanofluid over an elastic surface, as shown in Figure 1. The fluid contains two types of nanoparticles, i.e., nickel (Ni) and magnesium oxide (MgO).

The water is a base fluid that is electrically conducting, irrotational, and impinges on an elastic surface adjacent to a Darcian porous medium. The presence of an induced magnetic field is also considered since the magnetic Reynolds number is sufficiently large. Hall current and electrical polarization effects are neglected. The elastic surface is assumed to be porous, i.e., suction/injection are present. The velocity at the free stream is considered as $U_{f}=aX_{1}$ where a>0. The velocity at the surface is defined as $U_{s}=cX_{1}$ where c>0 corresponds to a stretching elastic surface, c<0 represents a contracting (shrinking) elastic surface, and c=0 indicates a stationary surface. The surface temperature is denoted by $T_{s}$ while the ambient temperature is denoted by $T_{\inf}$. The nanoparticles are of spherical shape, having zero agglomeration. In view of the above approximations, the governing equations in vectorial form can be defined as [36,37,38]:

(1) ∇⋅Π=0, ∇⋅U=0,

(2) $$U\cdot \nabla U-\frac{\mu_{e}}{4\pi \rho_{hnf}}\left(\Pi \cdot \nabla \right)\Pi =\upsilon_{hnf}\nabla^{2}U-\frac{1}{\rho_{hnf}}\nabla P-\frac{\upsilon_{hnf}}{k}U+\frac{g\left(T-T_{\inf}\right){\left(\rho \beta \right)}_{hnf}}{\rho_{hnf}},$$

(3) $$\nabla \times \left(U\times \Pi \right)=-\zeta \nabla^{2}\Pi ,$$

(4) $$\left(U\cdot \nabla \right)\;T=\frac{\kappa_{hnf}}{{\left(\rho C_{p}\right)}_{hnf}}\;\nabla^{2}T+\frac{S}{{\left(\rho C_{p}\right)}_{hnf}}\nabla U+\frac{\mu_{hnf}}{k{\left(\rho C_{p}\right)}_{hnf}}U^{2}+Q\left(T-T_{\inf}\right),$$

Here velocity field vector is designated by U, kinematic viscosity of hybrid nanofluid is denoted by $\upsilon_{hnf}$, induced magnetic field vector is represented by Π, thermal expansion coefficient is represented by $\beta_{hnf}$, the density of the hybrid nanofluid is denoted by $\rho_{hnf}$, the permeability of the porous medium is represented by *k*, the magnetic diffusivity is denoted by $\zeta =1/4\pi \mu_{e}\sigma_{hnf}$ (in which $\sigma_{hnf}$ denotes the electrical conductivity, the magnetic permeability parameter is represented by $\mu_{e}$), ${\left(C_{p}\right)}_{hnf}$ represents the specific heat capacity, $\kappa_{hnf}$ denotes the thermal conductivity, *T* is the temperature, pressure is represented by $P=\tilde{p}+\mu_{e}{\left|\Pi \right|}^{2}/8\pi$, and **S** is the viscous fluid stress tensor.

When using the boundary layer approximations, the governing Equations (1)–(4) are reduced to the following form in a two-dimensional system (*X*_1_, *X*_2_):

(5) $$\left.\begin{array}{l} \frac{\partial U_{2}}{\partial X_{2}}+\frac{\partial U_{1}}{\partial X_{1}}=0,\\ \frac{\partial \Pi_{2}}{\partial X_{2}}+\frac{\partial \Pi_{1}}{\partial X_{1}}=0, \end{array}\right\}$$

(6) $$\begin{array}{ll} U_{1}\frac{\partial U_{1}}{\partial X_{1}}+U_{2}\frac{\partial U_{1}}{\partial X_{2}} & =\upsilon_{hnf}\frac{\partial^{2}U_{1}}{\partial X_{2}^{2}}+\frac{\mu_{e}}{4\rho_{hnf}\pi }\left(\Pi_{1}\frac{\partial \Pi_{1}}{\partial X_{1}}+\Pi_{2}\frac{\partial \Pi_{1}}{\partial X_{2}}\right)\\ & +\left(U_{f}\frac{dU_{f}}{dX_{1}}-\frac{\mu_{e}\Pi_{f}}{4\pi \rho_{hnf}}\frac{d\Pi_{f}}{dX_{1}}\right)-\frac{\upsilon_{hnf}}{k}\left(U_{1}-U_{f}\right)+\frac{g{\left(\rho \beta \right)}_{hnf}}{\rho_{hnf}}\left(T-T_{\inf}\right), \end{array}$$

(7) $$U_{1}\frac{\partial \Pi_{1}}{\partial X_{1}}+U_{2}\frac{\partial \Pi_{1}}{\partial X_{2}}-\Pi_{1}\frac{\partial U_{1}}{\partial X_{1}}-\Pi_{2}\frac{\partial U_{1}}{\partial X_{2}}=\zeta \frac{\partial^{2}\Pi_{1}}{\partial X_{2}^{2}},$$

(8) $$U_{1}\frac{\partial T}{\partial X_{1}}+U_{2}\frac{\partial T}{\partial X_{2}}=\frac{\kappa_{hnf}}{{\left(\rho C_{p}\right)}_{hnf}}\frac{\partial^{2}T}{\partial X_{2}^{2}}+\frac{\mu_{hnf}}{{\left(\rho C_{p}\right)}_{hnf}}{\left(\frac{\partial U_{1}}{\partial X_{2}}\right)}^{2}+\frac{\mu_{hnf}}{{\left(\rho C_{p}\right)}_{hnf}k}{\left(U_{1}-U_{f}\right)}^{2}+Q\left(T-T_{\inf}\right),$$

In the above equations, $U_{1},U_{2}$ are the velocity components in the $X_{1},X_{2}$ directions (Cartesian coordinate system) where *X*_1_ is orientated along the elastic surface stretching direction and *X*_2_ is normal to it, $\Pi_{1},\Pi_{2}$ are the magnetic induction components and $\Pi_{f}$ represents the $X_{1}-$magnetic field towards the extremity of the boundary layer. The associated boundary conditions are defined at the wall and in the free stream as [39]:

(9) $$\left.\begin{array}{l} U_{1}=U_{s}+A_{u}\frac{\partial U_{1}}{\partial X_{2}},\;U_{2}=0,\;\Pi_{2}=\frac{\partial \Pi_{1}}{\partial X_{2}}=0,\;T=T_{s}+T_{u}\frac{\partial T}{\partial X_{2}}\;\mathrm{at}\;X_{2}\to 0,\\ U_{1}=U_{f}=aX_{1},\;U_{2}=0,\;\Pi_{1}=\Pi_{f}\left(X_{1}\right)=\Pi_{0}X_{1},\;T=T_{\inf}\;\mathrm{at}\;X_{2}\to \infty , \end{array}\right\}$$

Here $A_{u}$ represents the velocity slip, $T_{u}$ represents the thermal slip, $\Pi_{0}$ represents the magnetic field at infinity upstream.

The physical and thermodynamics properties of hybrid nanofluids are defined with the following relations:

- Density:
  (10) $$\rho_{hnf}=\rho_{1}^{np}\psi_{1}+\rho_{2}^{np}\psi_{2}+\rho_{f}(1-\psi_{hnf}),\psi_{hnf}=\psi_{1}+\psi_{2},$$
- Heat capacity:
  (11) $${\left(\rho C_{p}\right)}_{hnf}={\left(\rho C_{p}\right)}_{1}^{np}\psi_{1}+{\left(\rho C_{p}\right)}_{2}^{np}\psi_{2}+{\left(\rho C_{p}\right)}_{f}(1-\psi_{hnf}),\;$$
- Dynamic viscosity:
  (12) $$\mu_{hnf}=\frac{\mu_{f}}{{\left(1-\psi_{hnf}\right)}^{2.5}},$$
- Thermal conductivity:
  (13) $$\kappa_{hnf}=\kappa_{f}\times \left[\frac{2\kappa_{f}+\left(\frac{\psi_{1}\kappa_{1}^{np}+\psi_{2}\kappa_{2}^{np}}{\psi_{hnf}}\right)+2\left(\psi_{1}\kappa_{1}^{np}+\psi_{2}\kappa_{2}^{np}\right)-2\psi_{hnf}\kappa_{f}}{2\kappa_{f}+\left(\frac{\psi_{1}\kappa_{1}^{np}+\psi_{2}\kappa_{2}^{np}}{\psi_{hnf}}\right)-\left(\psi_{1}\kappa_{1}^{np}+\psi_{2}\kappa_{2}^{np}\right)+\psi_{hnf}\kappa_{f}}\right],$$
- Thermal expansion coefficient:
  (14) $${\left(\rho \beta \right)}_{hnf}={\left(\rho \beta \right)}_{f}(1-\psi_{hnf})+{\left(\rho \beta \right)}_{1}^{np}\psi_{1}+{\left(\rho \beta \right)}_{2}^{np}\psi_{2},\;$$
- Electric conductivity:
  (15) $$\sigma_{hnf}=\sigma_{f}\times \left[\frac{1+3\psi_{hnf}\left(\frac{\sigma_{hnp}}{\sigma_{f}}-1\right)}{\left(\frac{\sigma_{hnp}}{\sigma_{f}}+2\right)-\left(\frac{\sigma_{hnp}}{\sigma_{f}}-1\right)\psi_{hnf}}\right],\;\sigma_{hnp}=\frac{\psi_{1}\sigma_{1}^{np}+\psi_{2}\sigma_{2}^{np}}{\psi_{hnf}},$$

Here $\psi_{1}$ denotes the nanoparticle volume fraction of MgO nanoparticles, and $\psi_{2}$ denotes the nanoparticles volume fraction of Ni nanoparticles. The properties of the nanoparticles and base fluid (water) are given in Table 1.

## 3. Transformation of Mathematical Model

Let us introduce the following transformations to the mathematical model:

(16) $$\begin{array}{l} U_{1}=cX_{1}f^{\prime }\left(\eta \right),\;\Pi_{1}=\Pi_{0}X_{1}g^{\prime }\left(\eta \right),\\ U_{2}=-{\left(\upsilon_{f}c\right)}^{1/2}f\left(\eta \right),\\ \Pi_{2}=-{\left(\upsilon_{f}c\right)}^{1/2}\Pi_{0}g\left(\eta \right),\\ \eta =X_{2}{\left(c\upsilon_{f}^{-1}\right)}^{1/2},\;\vartheta =\frac{T-T_{\inf}}{T_{s}-T_{\inf}}, \end{array}$$

Substitution of Equation (16) into Equations (5)–(9) yields the following set of dimensionless coupled nonlinear ordinary differential equations:

(17) $$\frac{\Upsilon_{1}}{\Upsilon_{2}}f^{\prime \prime \prime }+ff^{\prime \prime }+\tau^{2}-{f^{\prime }}^{2}-\frac{M}{\Upsilon_{2}}\left(1-{g^{\prime }}^{2}+gg^{\prime \prime }\right)-\frac{\Upsilon_{1}}{\Upsilon_{2}}D_{a}\left(f^{\prime }-\tau \right)+G_{r}\frac{\Upsilon_{3}}{\Upsilon_{2}}\vartheta =0,$$

(18) $$\delta g^{\prime \prime \prime }-\Upsilon_{4}gf^{\prime \prime }+\Upsilon_{4}fg^{\prime \prime }=0,$$

(19) $$\frac{\Upsilon_{5}}{\Upsilon_{6}}\vartheta^{\prime \prime }+\frac{\Upsilon_{1}}{\Upsilon_{6}}E_{c}P_{r}{f^{\prime \prime }}^{2}+P_{r}\vartheta^{\prime }f+\frac{\Upsilon_{1}}{\Upsilon_{6}}E_{c}P_{r}D_{a}{\left(f^{\prime }-\tau \right)}^{2}+P_{r}q\vartheta =0.$$

The emerging reduced dimensionless boundary conditions are:

(20) $$\begin{array}{l} \mathrm{at}\;\eta \to 0:\\ f=0,\;f^{\prime }=1+\lambda f^{\prime \prime }\left(0\right),\;g^{\prime \prime }=0,\;g=0,\;\vartheta =1+\chi \vartheta^{\prime }\left(0\right),\\ \mathrm{at}\;\eta \to \infty :\\ \;f^{\prime }=\tau ,\;g^{\prime }=1,\;\vartheta =0, \end{array}$$

Here τ=a/c, $M\left(=\mu_{e}\Pi_{0}^{2}/4\pi \rho_{f}c^{2}\right)$ represents the magnetic parameter, $\lambda \left(=A_{u}{\left(c\upsilon_{f}^{-1}\right)}^{1/2}\right)$ represents the velocity slip parameter, $\chi \left(=T_{u}{\left(c\upsilon_{f}^{-1}\right)}^{1/2}\right)$ represents the thermal slip parameter, $G_{r}\left(=Gr/Re_{X_{1}}^{2}\right)$ the mixed convection parameter, $Gr\left(={\left(\rho \beta \right)}_{f}\left(T_{s}-T_{\inf}\right)X_{1}^{3}g/\rho_{f}\upsilon_{f}^{2}\right)$ is the thermal Grashof number, $E_{c}\left(=U_{s}^{2}/C_{p}\left(T_{s}-T_{\inf}\right)\right)$ is the Eckert number, $D_{a}\left(=\upsilon_{f}/ck\right)$ is the Darcy number for the porous medium, $\delta \left(=\zeta /\upsilon_{f}\right)$ is the reciprocal of magnetic Prandtl number, ReX1=X1Us/νf is the local Reynolds number [40], $P_{r}\left(={\left(\rho C_{p}\right)}_{f}\upsilon_{f}/\kappa_{f}\right)$ denotes the Prandtl number, and $q\left(=Q/{\left(\rho C_{p}\right)}_{f}\right)$ is the heat source/sink parameter.

The remaining parameters are as follows:

(21) $$\Upsilon_{1}=\frac{\mu_{hnf}}{\mu_{f}},\;\Upsilon_{2}=\frac{\rho_{hnf}}{\rho_{f}},\;\Upsilon_{3}=\frac{{\left(\rho \beta \right)}_{hnf}}{{\left(\rho \beta \right)}_{f}},\;\Upsilon_{4}=\frac{\sigma_{hnf}}{\sigma_{f}},\;\Upsilon_{5}=\frac{\kappa_{hnf}}{\kappa_{f}},\;\Upsilon_{6}=\frac{{\left(\rho C_{p}\right)}_{hnf}}{{\left(\rho C_{p}\right)}_{f}}.$$

The magnetic parameter, *M*, represents the ratio of kinetic to magnetic energy per unit volume, is related to the Hartmann number $H_{a}$. The magnetic parameter, *M*, Hartmann number, *H_a_*, Reynolds number, *Re*, and magnetic Reynolds number, *Re_m_*, are defined as:

(22) $$M=\frac{H_{a}^{2}}{ReRe_{m}},\;H_{a}=\mu_{e}\Pi_{0}\Gamma \sqrt{\frac{\sigma_{f}}{\mu_{e}}},\;Re=\frac{\Gamma \left(c\Gamma \right)}{\upsilon_{f}},\;Re_{m}=4\mu_{e}\sigma_{f}\Gamma \left(c\Gamma \right)\pi ,$$

In the above equation, Γ specifies the characteristic length of the elastic surface. For the magnetohydrodynamic boundary layer flows, M≤1, δ≥1—see Kumari et al. [41]. However, for electrically non-conducting flows, in the absence of a magnetic field M=0 and therefore Equation (18) is no longer required.

## 4. Engineering Quantities

The following are important physical quantities in engineering design for thermal coating flows, namely, the *skin friction coefficient* and the *Nusselt number*:

(23) $$C_{f}=\frac{S_{w}}{U_{s}^{2}\rho_{hnf}},\;N_{u}=\frac{Q_{w}X_{1}}{\left(T_{\inf}-T_{s}\right)\kappa_{f}},$$

Here $S_{w}$ represents the wall shear stress, and $Q_{w}$ represents the wall heat flux, which are defined as:

(24) $$S_{w}={\left.\mu_{hnf}\frac{\partial U_{1}}{\partial X_{2}}\right|}_{X_{2}=0},\;Q_{w}=-{\left.\kappa_{hnf}\frac{\partial T}{\partial X_{2}}\right|}_{X_{2}=0}.$$

Using the variables in Equation (16), the required expressions are:

(25) Cf=Υ1Υ2ReX1f″0, Nu=−Υ5ReX1ϑ′0,

## 5. Solution with MATLAB Numerical Shooting Method

A numerical shooting approach is utilized to solve the nonlinear differential Equations (17)–(19) with boundary conditions (20). MATLAB software has been used to generate all of the numerical solutions. The governing Equations (17)–(19) are first reduced to an initial value problem, which is expressed as follows:

(26) $$\left.\begin{array}{l} f=\hbar_{1}\\ f^{\prime }={\hbar^{\prime }}_{1}=\hbar_{2}\\ f^{\prime \prime }={\hbar^{\prime }}_{2}=\hbar_{3}\\ f^{\prime \prime \prime }={\hbar^{\prime }}_{3}=-\frac{\Upsilon_{2}}{\Upsilon_{1}}\hbar_{1}\hbar_{3}-\frac{\Upsilon_{2}}{\Upsilon_{1}}\tau^{2}+\frac{\Upsilon_{2}}{\Upsilon_{1}}\hbar_{2}^{2}-\frac{M}{\Upsilon_{1}}\left(\hbar_{5}^{2}-\hbar_{4}\hbar_{6}-1\right)+D_{a}\left(\hbar_{2}-\tau \right)-G_{r}\frac{\Upsilon_{3}}{\Upsilon_{1}}\hbar_{7} \end{array}\right\}$$

(27) $$\left.\begin{array}{l} g=\hbar_{4}\\ g^{\prime }={\hbar^{\prime }}_{4}=\hbar_{5}\\ g^{\prime \prime }={\hbar^{\prime }}_{5}=\hbar_{6}\\ g^{\prime \prime \prime }={\hbar^{\prime }}_{6}=\frac{\Upsilon_{4}}{\delta }\hbar_{3}\hbar_{4}-\frac{\Upsilon_{4}}{\delta }\hbar_{6}\hbar_{1} \end{array}\right\}$$

(28) $$\left.\begin{array}{l} \vartheta =\hbar_{7}\\ \vartheta^{\prime }={\hbar^{\prime }}_{7}=\hbar_{8}\\ \vartheta^{\prime \prime }={\hbar^{\prime }}_{8}=-\frac{\eta_{6}}{\eta_{5}}P_{r}\hbar_{8}\hbar_{1}-\frac{\eta_{1}}{\eta_{5}}E_{c}P_{r}\hbar_{3}^{2}-\frac{\Upsilon_{1}}{\Upsilon_{6}}E_{c}P_{r}D_{a}{\left(\hbar_{2}-\tau \right)}^{2}P_{r}q\hbar_{7} \end{array}\right\}$$

The boundary conditions (20) are formulated as:

(29) $$\begin{array}{l} \mathrm{at}\;\eta \to 0:\\ \hbar_{1}=0,\;\hbar_{2}=1+\lambda \hbar_{3},\;\hbar_{4}=0,\;\hbar_{6}=0,\;\hbar_{7}=1+\chi \hbar_{8},\\ \mathrm{at}\;\eta \to \infty :\\ \hbar_{2}=\tau ,\;\hbar_{5}=1,\;\hbar_{7}=0, \end{array}$$

In order to verify the MATLAB solutions, a comparison with previous studies is given in Table 2, Table 3 and Table 4. Excellent correlation is achieved for skin friction and the Nusselt number between the MATLAB solutions and Ali et al. [42] in Table 2. Further comparisons are given with Hassanien et al. [43], Salleh and Nazar [44] in Table 3, and Ali et al. [45] in Table 4. In all cases, very good agreement is demonstrated up to four decimal places, ensuring not only the validity of the current results but also the accuracy of the hybrid nanofluid results. Confidence in the MATLAB solutions is therefore justifiably high.

## 6. MATLAB Computational Results and Discussion

In this section, graphical and tabulated results are presented for the effects of all key parameters. The following parametric values have been chosen in order to carry out the computational formulation: $D_{a}=0.5;$ λ=0.2; χ=0.3;τ=2; δ=2; $E_{c}=0.5;$ $G_{r}=0.3;$ $\psi_{1}=0.2;$ $\psi_{2}=0.2;$ $P_{r}=6.96;$ M=0.2. The thermophysical properties of water, nickel (Ni), and magnesium oxide (MgO) nanoparticles have been given earlier in Table 1. Table 5 also contains the numerical data for the skin friction coefficient and the Nusselt number. It is evident that greater values of velocity slip parameter, the nanoparticle volume fraction of MgO, the heat source/sink parameter, and the Prandtl number all reduce the skin friction coefficient, whereas an increment in the Darcy parameter and the thermal Grashof number decreases skin friction. The Nusselt number decreases as the Darcy number, Eckert number, thermal Grashof number, thermal slip, the nanoparticle volume fraction of MgO and Ni, and Prandtl number increase, whereas it increases as the heat source/sink parameter and velocity slip increase.

**Figure 2:**
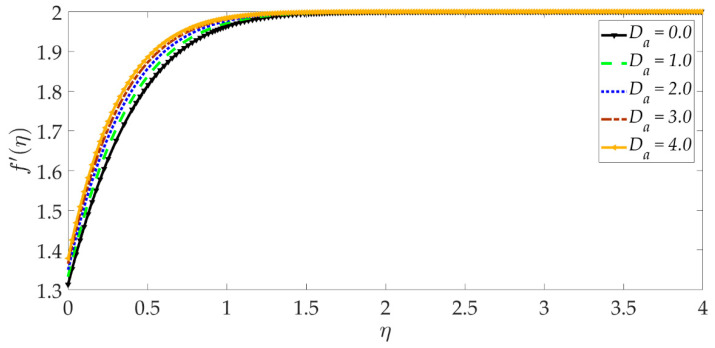
Consequences of Darcy number on velocity profile.

**Figure 3:**
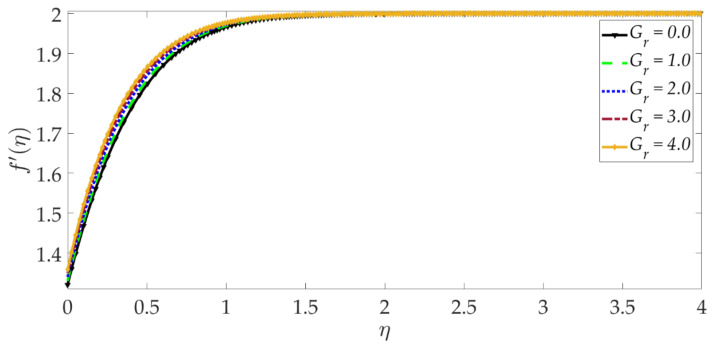
Consequences of thermal Grashof number on velocity profile.

**Figure 4:**
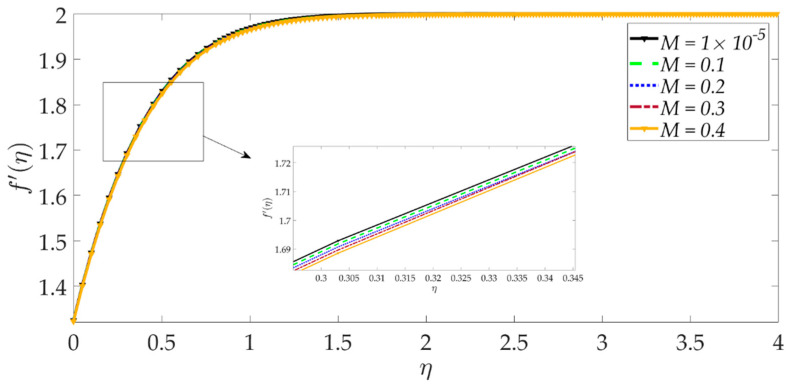
Consequences of magnetic parameter on velocity profile.

**Figure 5:**
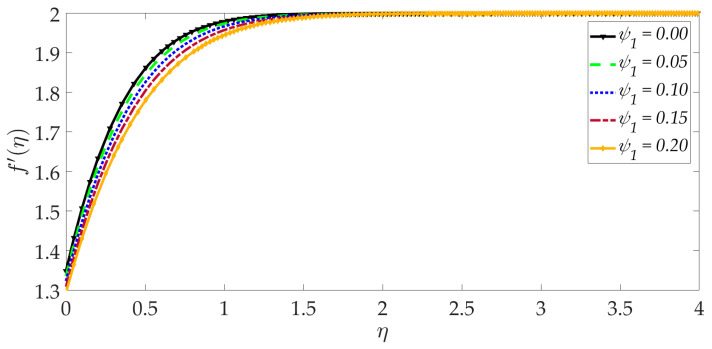
Consequences of nanoparticle volume fraction of MgO on velocity profile.

**Figure 6:**
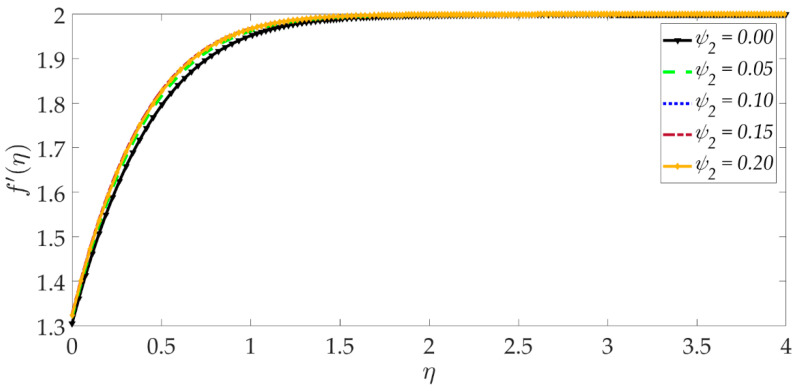
Consequences of nanoparticle volume fraction Ni on velocity profile.

**Figure 7:**
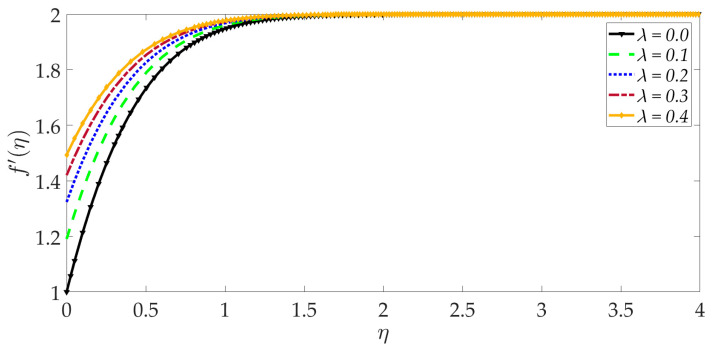
Consequences of velocity slip on velocity profile.

The variation of the velocity profile versus numerous values of various controlling parameters is shown in Figure 2, Figure 3, Figure 4, Figure 5, Figure 6 and Figure 7. Higher numerical values of the Darcy number $D_{a}$ enhance the velocity profile in the regime, as shown in Figure 2. The porous medium is assumed to be very sparsely packed, and therefore very high Darcy numbers are considered. The increase in permeability associated with a larger Darcy number reduces the Darcian impedance force, i.e., the resistance of solid matrix fibers to the percolating magnetic nanofluid, and this accelerates the flow leading to a depletion in momentum boundary layer thickness. With a higher Darcy number, the medium features lesser solid fibers. This assists in momentum development. A lower Darcy number, however, implies lower permeability, which results in a higher Darcian drag force and deceleration. Asymptotically smooth profiles are computed in the free stream, confirming the prescription of an adequately large infinity boundary condition in the MATLAB computations.

Figure 3 shows that elevation in the thermal Grashof number $G_{r}$ accentuates the velocity profile. The ratio of buoyancy to viscous forces is represented by the thermal Grashof number. Higher thermal Grashof numbers indicate that buoyancy force has a stronger role relative to the inhibitive viscous force. The thermal buoyancy force, $+G_{r}\frac{\Upsilon_{3}}{\Upsilon_{2}}\vartheta$ in Equation (17) is therefore amplified in magnitude, and this leads to acceleration. The impact of the magnetic parameter *M* on the velocity profile is shown in Figure 4. We can observe that the magnetic parameter slightly reduces the velocity at an intermediate distance from the wall. The magnetic force term $-\frac{M}{\Upsilon_{2}}\left(1-g^{\prime 2}+gg^{\prime \prime }\right)$ in Equation (17) inhibits the boundary layer flow and increases the momentum boundary layer thickness slightly. Figure 5 shows that when the volume fraction of MgO nanoparticles $\psi_{1}$ increases, the velocity profile decreases dramatically. Greater doping of the nanofluid with MgO nanoparticles, therefore, decelerates the flow and increases momentum boundary layer thickness. However, the behavior of a unitary nanofluid, i.e., the profile $\psi_{1}=0$ (where only Ni nanoparticles are present), achieves the maximum velocity and minimal momentum boundary layer thickness. On the other hand, with increasing volume percentage of Ni nanoparticles $\psi_{2}$, the velocity is enhanced, as seen in Figure 6. The unitary nanofluid $\psi_{2}=0$ (for which MgO nanoparticles are absent) in this case produces the minimal velocity and maximum momentum boundary layer thickness. Figure 7 indicates that increasing the values of the velocity slip parameter enhances the fluid motion since greater momentum is generated at the wall, which assists the flow; the momentum boundary layer thickness is reduced with greater velocity slip. For the case λ=0 when hydrodynamic slip is absent, the velocity is minimal, and momentum boundary layer thickness is maximum.

**Figure 8:**
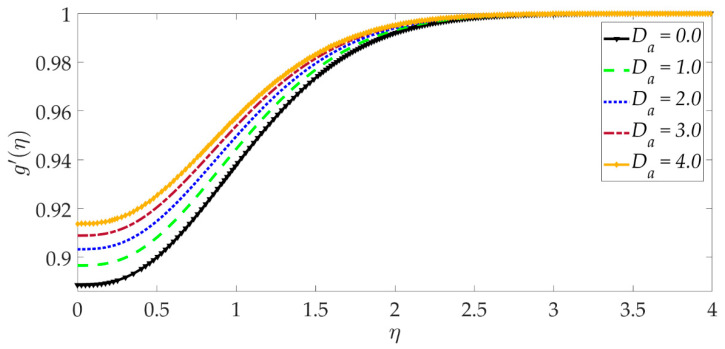
Consequences of Darcy number on induced magnetic field profile.

**Figure 9:**
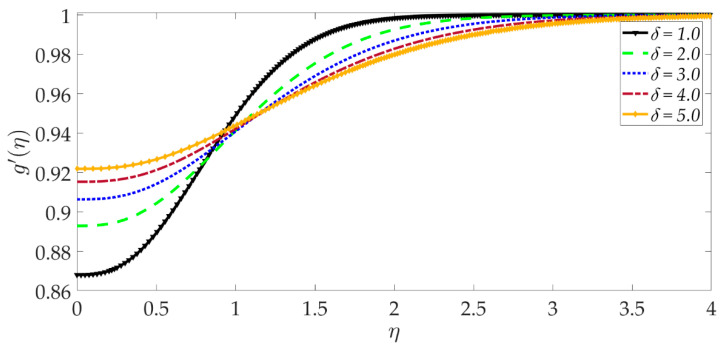
Consequences of reciprocal magnetic Prandtl number on induced magnetic field profile.

**Figure 10:**
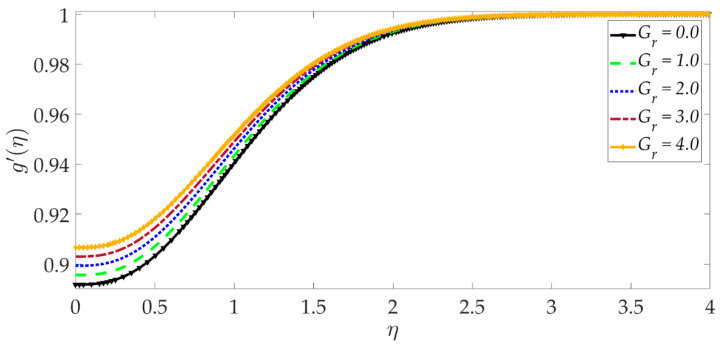
Consequences of thermal Grashof number on induced magnetic field profile.

**Figure 11:**
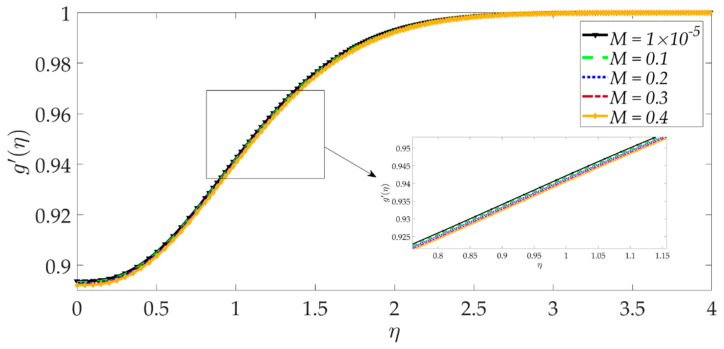
Consequences of magnetic parameter on induced magnetic field profile.

**Figure 12:**
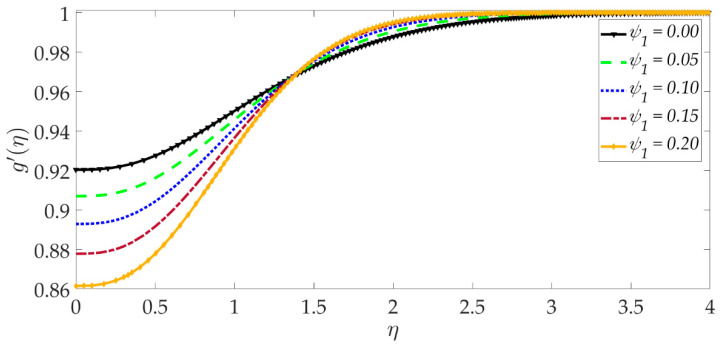
Consequences of nanoparticle volume fraction MgO on induced magnetic field profile.

**Figure 13:**
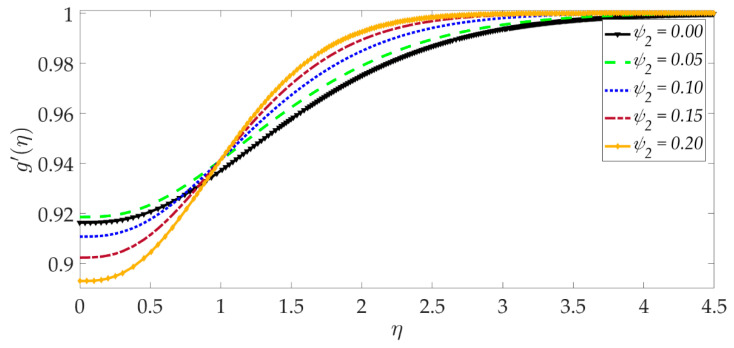
Consequences of nanoparticle volume fraction Ni on induced magnetic field profile.

**Figure 14:**
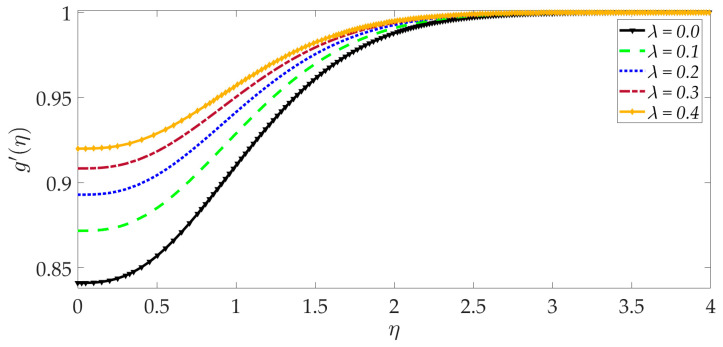
Consequences of velocity slip on induced magnetic field profile.

The influence of selected parameters on the induced magnetic field is depicted in Figure 8, Figure 9, Figure 10, Figure 11, Figure 12, Figure 13 and Figure 14. As seen in Figure 8, raising the numerical values of the Darcy number $D_{a}$ increases the induced magnetic field $g^{\prime }$ magntiudes dramatically. Although the Darcian body force does not appear explicitly in the magnetic induction Equation (18), via coupling with the momentum Equation (17), i.e., the terms $-\Upsilon_{4}gf^{\prime \prime }+\Upsilon_{4}fg^{\prime \prime }$, the Darcy number influences indirectly the magnetic induction field. A sigmoidal topology is observed from the wall (elastic surface) to the free stream. Magnetic boundary layer thickness is increased significantly with increment in Darcy number (i.e., higher permeability of the porous medium). In Figure 9, we can see that increasing the reciprocal of magnetic Prandtl number δ, i.e., decreasing magnetic Prandtl number, boosts the induced magnetic field at first; however, further from the elastic surface after η>1, the tendency reverses, and there is a depletion in magnetic induction. Magnetic Prandtl number expresses the relative rate of momentum (viscous) diffusion to magnetic diffusion in the regime. This parameter significantly modifies the induced magnetic field distribution in the regime, but the response is dependent on location from the elastic surface. The thermal Grashof number $G_{r}$ strongly enhances the induced magnetic field profile, as shown in Figure 10. For the case of forced convection, *G_r_* = 0 and thermal buoyancy effects vanish. The magnetic induction is minimized for this case, as is the magnetic boundary layer thickness. Overall thermal buoyancy is assistive to the induced magnetic field and increases magnetic boundary layer thickness on the elastic surface. In Figure 11, we can see that the magnetic parameter *M* has a depleting effect on the induced magnetic field profile. Larger *M* values suppress the magnetic boundary layer thickness. In Figure 12 and Figure 13, we can see that the volume percentage of MgO and Ni $\left(\psi_{1},\psi_{2}\right)$ nanoparticles initially reduces the induced magnetic field values closer to the elastic surface (wall); however, further into the boundary layer regime, transverse to the wall, the trend is opposite, and there is a clear enhancement in magnitudes of the induced magnetic field, which is sustained into the free stream. Furthermore, in these plots, the special cases of unitary (single nanoparticle) nanofluids correspond to $\psi_{1}=0\;\mathrm{or}\;\psi_{2}=0$. The effects of the hydrodynamic (velocity) slip parameter on the induced magnetic field profile are depicted in Figure 14. It is apparent that the induced magnetic profile is greatly boosted with an increment in the slip parameter. The coupling of the momentum and induced magnetic field Equations (17) and (18) again enables the momentum slip to influence the magnetic induction via the boundary condition,$\;f^{\prime }=1+\lambda f^{\prime \prime }\left(0\right)$ in Equation (20). For the case λ=0 for which wall momentum slip is absent, the induced magnetic field is minimized, and the magnetic boundary layer thickness is also minimal.

**Figure 15:**
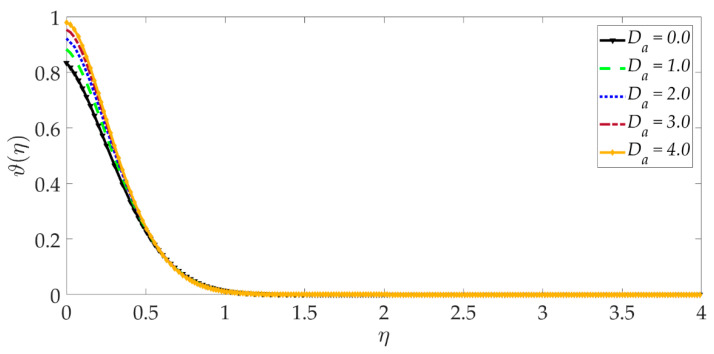
Consequences of Darcy number on temperature profile.

**Figure 16:**
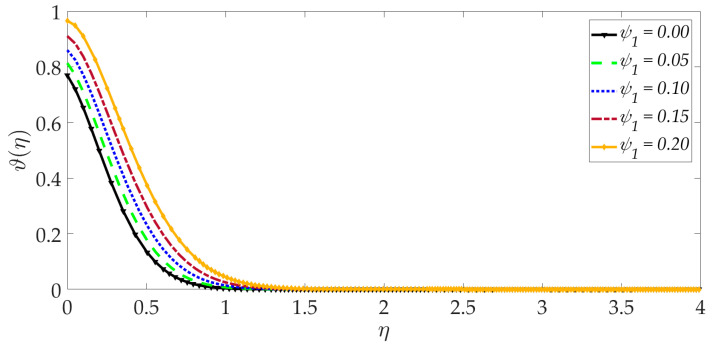
Consequences of nanoparticle volume fraction MgO on temperature profile.

**Figure 17:**
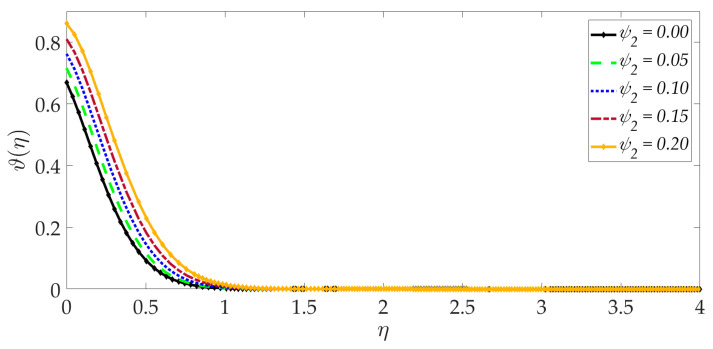
Consequences of nanoparticle volume fraction Ni on temperature profile.

**Figure 18:**
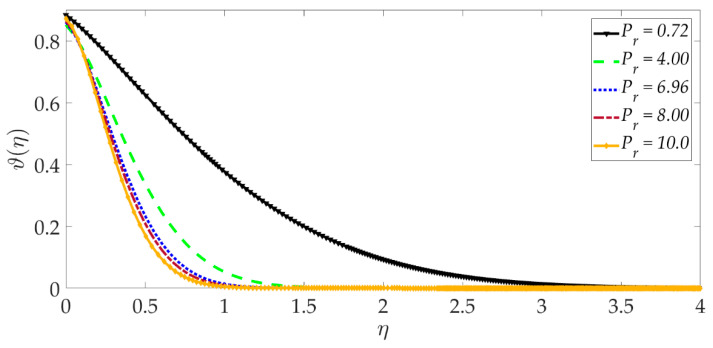
Consequences of Prandtl number on temperature profile.

**Figure 19:**
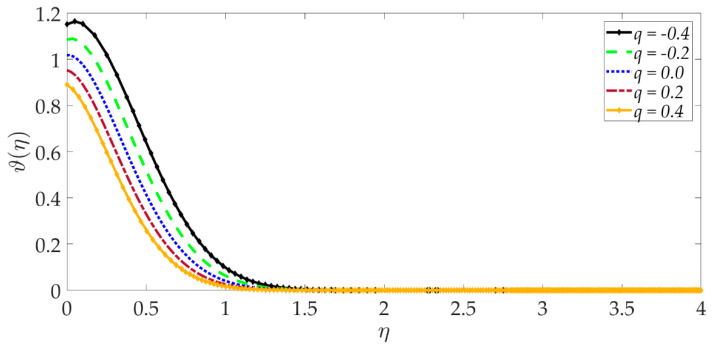
Consequences of heat source/sink parameter on temperature profile.

**Figure 20:**
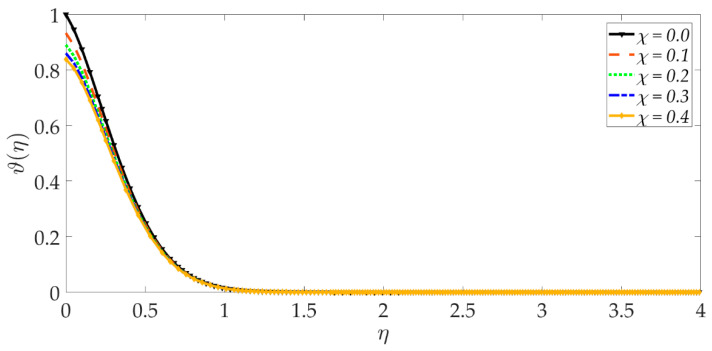
Consequences of thermal slip parameter on temperature profile.

Figure 15, Figure 16, Figure 17, Figure 18, Figure 19 and Figure 20 illustrate the evolution in temperature profiles for selected parameters. It can be shown in Figure 15 that with elevation in Darcy number, there is a strong increment in the temperature profile and thermal boundary layer thickness. The prominent modification, i.e., heating, is near the elastic surface (wall), and further, the effect decays when η>0.5, which is sustained into the freestream. It can be deduced from Figure 16 and Figure 17 that increasing the nanoparticle volume percentage of MgO and Ni nanoparticles $\left(\psi_{1},\psi_{2}\right)$ results in a significant and uniform enhancement in the temperature profile and thermal boundary layer thickness. Greater doping with MgO and Ni nanoparticles, therefore, achieves a desirable thermal elevation in particular close to the wall (elastic surface). Figure 18 shows that temperatures are suppressed and, therefore, also, the thermal boundary layer thickness is reduced with increment in Prandtl number $P_{r}$. Higher values of the Prandtl number indicate that momentum diffusivity is becoming more dominant over thermal diffusivity, and the net effect is a suppression of thermal diffusion. This cools the regime and depletes the thickness of the thermal boundary layer. The impact of the heat source/sink parameter (*q*) on the temperature profile is depicted in Figure 19. It should be noted that increasing heat generation results in a boost in temperatures, whereas increasing heat sink induces the opposite effect. Thermal boundary layer thickness is therefore also modified with either heat generation (source) or absorption (sink). The case q=0 corresponds to the absence of the heat source/sink parameter and produces intermediate values of temperature between the heat source and sink cases. Finally, Figure 20 demonstrates that an increment in thermal slip has the effect of decreasing the temperature magnitudes and also the thickness of the thermal boundary layer. The thermal slip parameter, i.e., χ, arises in the modified wall thermal boundary condition $\vartheta =1+\chi \vartheta^{\prime }\left(0\right),$ in Equation (20). It creates a thermal jump effect which delays the heat transfer from the wall to the boundary layer regime on the elastic surface. This leads to a reduction in temperatures, i.e., the cooling effect.

## 7. Conclusions

A mathematical model has been derived for the steady, incompressible, magnetohydrodynamic (MHD) stagnation flow of an electrically conducting hybrid nanofluid (comprising magnesium oxide and nickel nanoparticles in water base fluid) along an elastic surface embedded in a porous medium. The magnetic Reynolds number is sufficiently large to invoke magnetic induction effects. Darcy’s law is adopted for the porous medium. Wall velocity slip, thermal slip, heat source/sink, and viscous dissipation are also incorporated in the model. The transformed dimensionless nonlinear ordinary differential boundary layer equations for momentum, induced magnetic field, and energy with appropriate boundary conditions are solved with Matlab software using a shooting method. A detailed comparison has been presented with previous studies from the literature for skin friction and Nusselt number in order to verify the accuracy and convergence of the present Matlab solutions. The primary findings of the current investigation may be summarized as follows:

- With increasing nanoparticle volume fraction of nickel, velocity slip, thermal Grashof number, and the Darcy number, there is a strong acceleration in the flow and reduction in momentum boundary layer thickness.
- With the elevation in the magnetic parameter and the nanoparticle volume fraction of magnesium oxide, there is a deceleration in the flow and an increase in momentum boundary layer thickness.
- An increment in nanoparticle volume fraction of both MgO and Ni boosts the temperature and thermal boundary layer thickness.
- Elevation in Darcy number, velocity slip, and thermal Grashof number generally enhance the induced magnetic field and increase magnetic boundary layer thickness.
- An increment in both MgO and Ni-nanoparticle volume fractions increases magnetic induction primarily near the elastic surface (wall).
- With an increase in the reciprocal of magnetic Prandtl number (i.e., decrease in magnetic diffusion rate relative to viscous diffusion rate), initially, there is a suppression in the induced magnetic field near the wall; however, further from the wall, this trend is reversed.
- The temperature profile and thermal boundary layer thickness are boosted with increment in Darcy number and both MgO and Ni nanoparticle volume fractions.
- Increasing the Prandtl number, the heat sink parameter, and the thermal slip all have the effect of decreasing the thickness of the thermal boundary layer and the temperature magnitudes.
- Matlab shooting quadrature is found to be very accurate for solving magnetized hybrid nanofluid coating flows and achieves very good convergence characteristics.

The present study has considered *Newtonian* flow behavior. Future investigations may address the *rheology* of hybrid nanofluids (e.g., viscoelastic models [46,47]) and also consider alternative nanoparticles, e.g., combinations of metallic (gold, silver, Zinc oxide, etc.) and carbon-based (diamond, graphite, etc.) [48,49]. Efforts in these directions are also beneficial to solar energy coating manufacturing fluid dynamics and will be reported imminently.

## Figures and Tables

**Table 1:** Thermophysical properties of water, magnesium oxide and nickel nanoparticles.

Physical Properties	Water	Nickel (Ni)	Magnesium Oxide (MgO)
$\rho \left(\mathrm{kg}/m^{3}\right)$	997.1	8908	3580
$\kappa \left(W/\mathrm{mK}\right)$	0.613	91	45
$C_{p}\left(J/\mathrm{kgK}\right)$	4179	445	955
$\beta \left(1/K\right)$	0.00021	0.0000134	0.0000336
$\sigma \left(S/m\right)$	0.05	1.7 × 10^7^	2.6 × 10^−6^

**Table 2:** A numerical comparison of skin friction and Nusselt number by considering the following values: $D_{a}=0;\;\lambda =0;\;\chi =0;\;\tau =0;\;E_{c}=0;\;G_{r}=0;\;\psi_{1}=0;\;\psi_{2}=0;\;q=0$.

	$C_{f}$	$N_{u}$ $\left(P_{r}=0.72,10\right)$	$C_{f}$	$N_{u}$ $\left(P_{r}=0.72,10\right)$
δ	Ali et al. [42]		Present Results	
10^2^	0.9914	(0.4713, 2.30818)	0.999654056	(0.463845643, 2.308119015)
10^3^	0.9993	(0.4639, 2.30809)	0.999975012	(0.463616023, 2.308031227)
10^4^	0.9999	(0.4632, 2.30808)	1.000004856	(0.463594549, 2.308022703)
10^5^	1.0000	(0.4632, 2.30808)	1.000007819	(0.463592416, 2.308021857)
M	$\delta =10^{4}$			
0.01	0.9993	(0.4639, 2.30809)	0.999975236	(0.463616264, 2.308024896)
0.05	0.9970	(0.4663, 2.30812)	0.999889007	(0.465078584, 2.308044206)
0.10	0.9945	(0.4687, 2.30817)	0.996099015	(0.467058435, 2.308027518)
0.15	0.9922	(0.4707, 2.30822)	0.992392113	(0.472321992, 2.307876202)

**Table 3:** A numerical comparison of Nusselt number by considering the following values: $D_{a}=0;\;\lambda =0;\;\chi =0;\;\tau =0;\;E_{c}=0;\;G_{r}=0;\;\psi_{1}=0;\;\psi_{2}=0;\;q=0$.

$P_{r}$	τ=0,M=0			
	Ali et al. [42]	Hassanien et al. [43]	Salleh and Nazar [44]	Present Results
0.72	0.4632	0.46325	0.46317	0.463592073
10	2.3081	2.30801	2.30821	2.308006492

**Table 4:** A numerical comparison of skin friction and Nusselt number by considering the following values: $D_{a}=0;\;\lambda =0;\;\chi =0;\;\tau =0;\;E_{c}=0;\;G_{r}=0;\;\psi_{1}=0;\;\psi_{2}=0;\;q=0$.

	τ=3,M=1			
	Skin Friction		Nusselt Number	
$P_{r}$	Ali et al. [45]	Present Results	Ali et al. [45]	Present Results
0.07			0.33814	0.338140112
0.72	4.52158	4.521582090	0.97240	0.972402233
0.5			0.82748	0.827484508
2.0			1.52147	1.521468063
6.8			2.59780	2.597812407
10			3.07902	3.079050771
***M***	$\tau =3,P_{r}=0.72$			
0.1	4.70928	4.709283867	0.97902	0.979021615
0.5	4.62764	4.627634204	0.97617	0.976172800
1.0	4.52158	4.521582090	0.97240	0.972402233
2.0	4.29431	4.294313656	0.96405	0.964045689
$\tau =0.5,P_{r}=0.72$				
0.10	−0.57595	−0.575949681	0.59171	0.591705825
0.15	−0.50938	−0.509403808	0.60207	0.602061052
0.20	−0.40717	−0.407547217	0.61811	0.618079173

**Table 5:** Numerical results of skin friction and Nusselt number against all the leading parameters.

δ	M	$P_{r}$	λ	$D_{a}$	$E_{c}$	$G_{r}$	χ	$\psi_{1}$	$\psi_{2}$	q	$C_{f}$	$N_{u}$
1	0.2	6.96	0.2	0.5	0.5	0.3	0.3	0.2	0.2	0.5	1.386342185	1.046697391
2											1.387113826	1.046037055
3											1.387580951	1.045443513
	0.01										1.391446326	1.037575829
	0.1										1.389402333	1.041569479
	0.2										1.387113826	1.046037055
		5									1.387916242	1.093391191
		6.96									1.387113826	1.046037055
		8									1.386806321	1.015829923
			0								1.983946596	−1.847797617
			0.1								1.636922168	−0.007837483
			0.2								1.387113826	1.046037055
				0							1.343075838	1.240588255
				1							1.427934896	0.874197208
				2							1.501620476	0.584125598
					0.1						1.381818954	2.951411753
					0.2						1.383143556	2.475622258
					0.3						1.384467393	1.999464353
						0					1.374362440	1.050348820
						1					1.416662936	1.035561579
						2					1.458421488	1.019592211
							0				1.388693182	1.931909381
							0.1				1.387935006	1.506589469
							0.2				1.387450377	1.234763174
								0			1.178305552	1.329401404
								0.1			1.387113826	1.046037055
								0.2			1.699319889	0.329337847
									0		1.698735776	1.423814942
									0.1		1.599004165	1.103502632
									0.2		1.699319889	0.329337847
										−0.2	1.706475625	−1.563796173
										0	1.704165102	−1.016305551
										0.2	1.702063147	−0.467389070

## Data Availability

Not applicable.

## Nomenclature

**Symbols**	**Names**
$U_{f}$	Velocity at the free stream
$U_{s}$	Velocity at the surface
$T_{\inf}$	Ambient temperature
$T_{s}$	Surface temperature
U	Velocity field vector
P	Pressure
${\left(C_{p}\right)}_{hnf}$	Specific heat
**S**	Stress tensor
*K*	Permeability of the porous medium
$X_{1},X_{2}$	Cartesian coordinate system
$U_{1},U_{2}$	Velocity components
$A_{u}$	Velocity slip
$T_{u}$	Thermal slip
M	Magnetic parameter
$G_{r}$	Mixed convection parameter
Gr	Thermal Grashof number
$E_{c}$	Eckert number
$D_{a}$	Darcy number
ReX1	Local Reynolds number
$P_{r}$	Prandtl number
q	Heat source/sink parameter
*H_a_*	Hartmann number
*Re_m_*	Magnetic Reynolds number
$S_{w}\;$	Wall shear stress
$Q_{w}\;$	Wall heat flux
**Greek Symbols**	
$\upsilon_{hnf}$	Kinematic viscosity
Π	Induced magnetic field vector
$\beta_{hnf}$	Thermal expansion coefficient
$\rho_{hnf}$	Density of the hybrid nanofluid
ζ	Magnetic diffusivity
$\sigma_{hnf}$	Electrical conductivity
$\mu_{e}$	Magnetic permeability parameter
$\Pi_{1},\Pi_{2}$	Magnetic induction components
$\Pi_{0}$	Magnetic field at infinity upstream
$\psi_{1}$	Nanoparticle volume fraction of MgO nanoparticles
$\psi_{2}$	Nanoparticle’s volume fraction of Ni
λ	Velocity slip parameter
χ	Thermal slip parameter
δ	Reciprocal of magnetic Prandtl number
Γ	Characteristic length of the elastic surface
**Subscripts and Abbreviations**	
MgO	Magnesium oxide
Ni	Nickel
*hnf*	Hybrid nanofluid
*nf*	Nanofluid
NPs	Nanoparticles
